# Relationship between the Direction of Ophthalmic Artery Blood Flow and Ocular Microcirculation before and after Carotid Artery Stenting

**DOI:** 10.1155/2016/2530914

**Published:** 2016-12-14

**Authors:** Masashi Ishii, Morito Hayashi, Fumihiko Yagi, Kenichiro Sato, Goji Tomita, Satoshi Iwabuchi

**Affiliations:** ^1^Department of Neurosurgery, Toho University Ohashi Medical Center, Tokyo, Japan; ^2^Department of Ophthalmology, Toho University Ohashi Medical Center, Tokyo, Japan

## Abstract

When internal carotid artery stenosis is accompanied by ocular ischemic syndrome, intervention is recommended to prevent irreversible visual loss. In this study, we used laser speckle flowgraphy to measure the ocular microcirculation in the optic nerve head before and after carotid artery stenting (CAS) of 40 advanced internal carotid stenosis lesions from 37 patients. The aim was to investigate the relationship between ocular microcirculation and the direction of ophthalmic artery blood flow obtained by angiography. We found that there was a significant increase in blood flow after CAS (*P* = 0.003). Peak systolic velocity as an indicator of the rate of stenosis was also significantly higher in the group with retrograde/undetected flow of the ophthalmic artery than in the group with antegrade flow (*P* = 0.002). In all cases where retrograde flow of the ophthalmic artery was observed before stenting, the flow changed to antegrade after stenting. Through the use of laser speckle flowgraphy, this study found that CAS can improve ocular microcirculation. Furthermore, while patients displaying retrograde flow of the ophthalmic artery before stenting have a poor prognosis, CAS corrected the flow to antegrade, suggesting that visual loss can be prevented by improving the ocular microcirculation.

## 1. Introduction

The ophthalmic artery is the first intradural branch of the carotid artery and its function is intricately correlated with the ocular microcirculation and carotid artery blood flow [1–3]. Visual function disorder symptoms accompanying carotid artery stenosis are termed ocular ischemic syndrome (OIS) [4–6]. In its acute state, OIS manifests as amaurosis fugax and retinal artery occlusion, whilst in its in chronic state, OIS manifests as retinopathy and rubeotic or neovascular glaucoma. Revascularization surgery such as carotid endarterectomy and carotid artery stenting (CAS) is performed to treat advanced carotid artery stenosis. Ophthalmic artery Doppler waveforms and fluorescent fundus angiography have been the most widely employed techniques for evaluating the ocular microcirculation [7–10]. However, in recent years, more consideration has been given to laser speckle flowgraphy (LSFG) as a noninvasive method for quantitatively measuring ocular microcirculation. Numerous studies have reported on the effectiveness of LSFG [11–13].

In this study, we have evaluated the effectiveness of CAS in correcting ocular microcirculation by using LSFG to measure the blood flow in the optic nerve head (ONH) before and after CAS. Furthermore, as retrograde blood flow is often observed in advanced carotid artery stenosis and carotid artery occlusion, angiography was used to investigate the correlation between the direction of ophthalmic artery blood flow and changes in the ocular microcirculation.

## 2. Materials and Methods

### 2.1. Patient Recruitment

The study was conducted from September 2012 to March 2016 at the Toho University Ohashi Medical Center, Tokyo, Japan. Thirty-seven advanced carotid artery stenosis patients (31 males/6 females) who were given LSFG before and after CAS were included in the study, representing a total of 40 lesions. Patient age ranged from 60 to 89 years (mean age 76.1 ± 6.7 years). Twenty-five of the lesions were located in the right eye and 15 in the left eye. Eleven cases were symptomatic, of which four had visual symptoms (three cases with amaurosis fugax, one case with retinal artery occlusion). This study was conducted in accordance with the ethical principles of the Declaration of Helsinki and was approved by the ethics review committee of Toho University Ohashi medical Center (2012-84).

### 2.2. Measurement of Ocular Microcirculation

**Figure 1:**
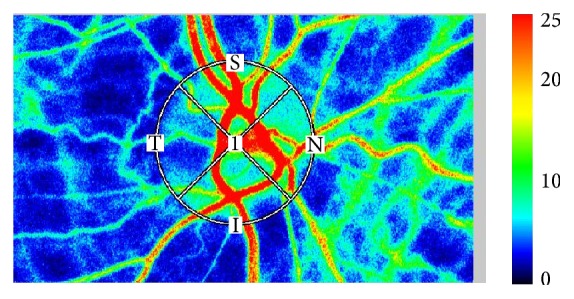
We used LSFG-NAVI to evaluate the ocular microcirculation, with three tests performed in each eye. We measured all MBR in the optic nerve head. In the color-coded maps, red indicates high blood flow and blue indicates low blood flow.

**Figure 2:**
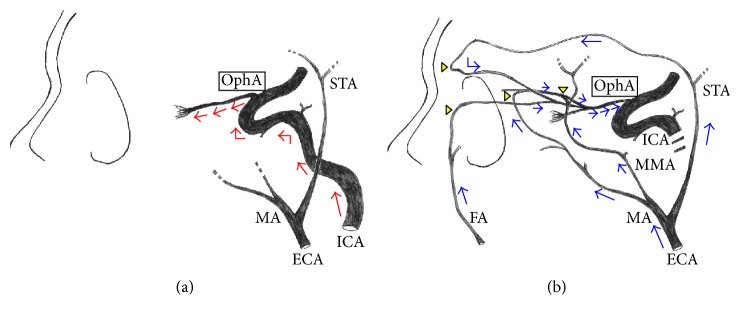
(a) Usually, the ophthalmic artery receives blood flow from the internal carotid artery, which means that the ophthalmic artery is visualized antegradely from the bifurcation from the internal carotid artery (red arrows). (b) However, when advanced stenotic lesions are seen in the internal carotid artery, the ophthalmic artery instead receives blood flow from the external carotid artery; thus, it is visualized retrogradely from the peripheral side via the multiple anastomoses (arrow heads) with the external carotid artery (blue arrows). ^*∗*^OphA, ophthalmic artery; ICA, internal carotid artery; ECA, external carotid artery; STA, superficial temporal artery; MA, maxillary artery; FA, facial artery; MMA, middle meningeal artery.

**Figure 3:**
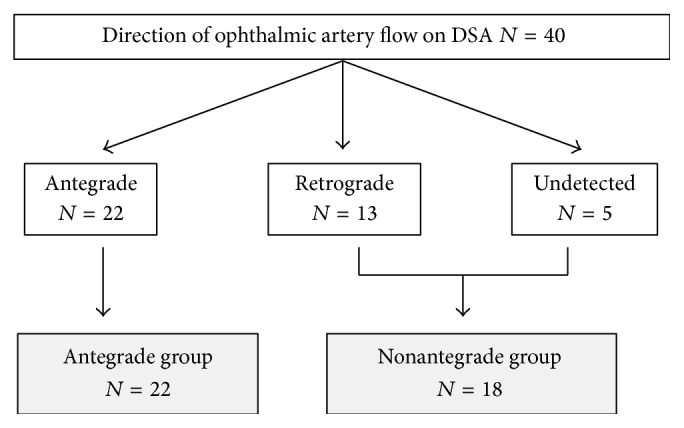
Classification of study participants by direction of ophthalmic artery blood flow on digital subtraction angiography (DSA). All the participants were classified into 3 groups and recategorized 2 groups for the purpose of this study.

LSFG-NAVI (Softcare Co. Ltd., Fukuoka, Japan) was used to evaluate the ocular microcirculation, with three tests performed in each eye. During the LSFG procedure, systemic blood pressure was measured to assess if there was any physiological response. Mean blur rate (MBR) was measured at the area of microcirculation in the ONH (Figure 1) and was defined as the retinochoroidal blood flow parameter, with the average of three measurements taken. LSFG was performed within 3 months prior to CAS and within 1 month following CAS. Rate of stenosis in the carotid artery was measured using ultrasound and evaluated by peak systolic velocity (PSV) [14, 15]. The direction of the ophthalmic artery blood flow was determined using angiography prior to CAS (Figure 2) and the findings were classified into three groups: antegrade (*N* = 22), retrograde (*N* = 13), and undetected (*N* = 5). For the purpose of this study, these groups were recategorized; antegrade group (*N* = 22) and nonantegrade group (*N* = 18; retrograde and undetected) (Figure 3).

### 2.3. Statistical Analysis

SPSS version 21.0 was used for statistical analysis (IBM SPSS Inc., Chicago, IL, USA). Wilcoxon signed-rank test was used to compare the MBR and mean arterial pressure before and after CAS. The Chi-square test was used to compare the rate of stenosis in the antegrade and nonantegrade groups before and after CAS and the Mann–Whitney *U* test was used to compare the MBR and PSV between these groups. Statistical significance was accepted as *P* < 0.05.

## 3. Results

### 3.1. Change in the Mean Blur Rate before and after Carotid Artery Stenting

**Figure 4:**
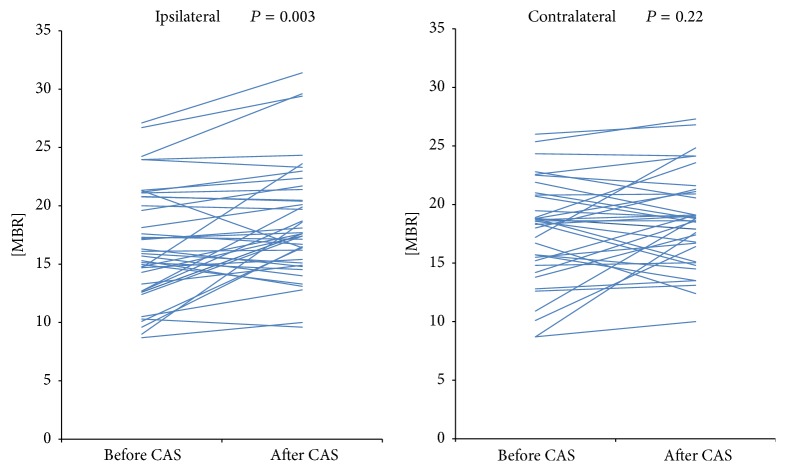
Change in the mean blur rate (MBR) before and after carotid artery stenting (CAS). The MBR on the operated side showed a statistically significant increase following CAS (*P* = 0.003).

The MBR on the operated side showed a statistically significant increase following CAS (*P* = 0.003), whilst there was no difference on the contralateral side (Figure 4). Mean arterial pressure, taken simultaneous to the procedure, showed a significant decrease after surgery (*P* < 0.001) (Table 1).

### 3.2. Direction of Ophthalmic Artery Blood Flow before and after Carotid Artery Stenting

**Figure 5:**
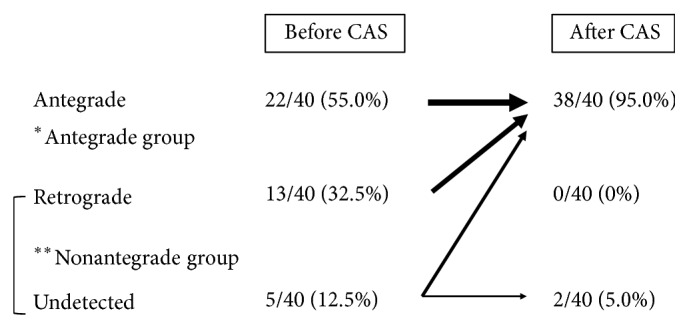
Direction of ophthalmic artery blood flow before and after CAS. In our investigation, all cases in the retrograde group and 3 cases in the undetected group had successfully recovered antegrade flow after CAS.

Angiography taken before CAS showed antegrade flow in 22 lesions and nonantegrade flow in 18 lesions (13 lesions in retrograde group and five lesions in undetected group). Patients in the antegrade group were significantly older than patients in the nonantegrade group (*P* = 0.02). The rate of carotid artery stenosis was significantly higher in the nonantegrade group, along with the PSV (*P* = 0.002) (Table 2). Following CAS, angiography determined that 38 patients were in the antegrade group and two patients were in the nonantegrade group (Figure 5).

### 3.3. Direction of Ophthalmic Artery Blood Flow and Changes in the Mean Blur Rate

**Figure 6:**
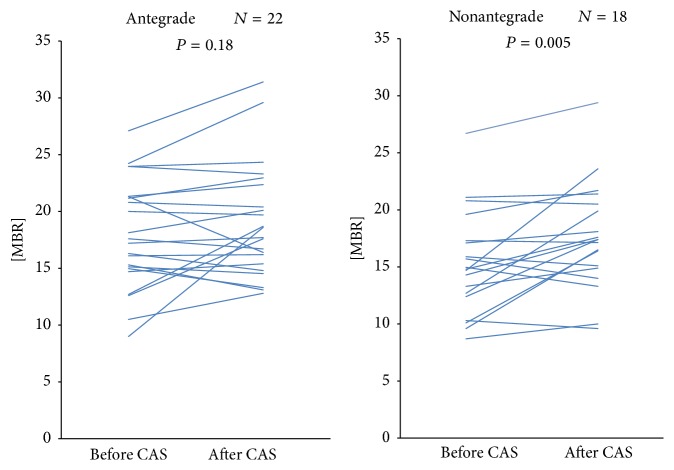
Change in the MBR before and after CAS in the antegrade and nonantegrade groups. There was the significant rise in the MBR in the nonantegrade group (*P* = 0.005).

There was no significant difference in the MBR between the antegrade and nonantegrade groups before and after CAS (Table 2). Within the antegrade group alone, there was a rise in the MBR after CAS but this was not significant (*P* = 0.18). However, there was a significant increase in the MBR after CAS in the nonantegrade group (*P* = 0.005) (Figure 6).

## 4. Discussion

Blood is supplied to the retina through the central retinal artery and the short posterior ciliary artery that branches from the first intradural branch of the carotid artery. Advanced stenosis in the carotid artery will therefore hemodynamically cause hypoperfusion to the eye, leading to ocular ischemic syndrome (OIS) [16]. Progression of OIS can lead to an irreversible decrease in visual acuity and subsequent loss of vision, which can be prevented through early detection and intervention [4, 17]. In patients with advanced internal carotid artery (ICA) stenosis accompanying OIS, improvements in visual function after revascularization surgery have been reported [18, 19]. Improvements in ocular circulation following evaluation by ophthalmic artery Doppler waveform or fluorescent fundus angiography have also been reported [9, 18, 19]. While ophthalmic artery Doppler waveform enables evaluation of blood flow direction and speed, there is a risk of tissue damage because of the thermal effects of the ultrasound [19]. Speed and technical sophistication are also crucial when applying this test. Problems are also evident with fluorescent fundus angiography, as it is highly biased by individual physiological components and has poor repeatability [9].

Compared with these conventional test methods, LSFG requires no contrast agent, allows for noninvasive, quantitative measurement of retinochoroidal blood flow, and demonstrates good repeatability. LSFG uses laser speckle methodology to analyze in a two-dimensional map the measurement of the ocular fundus blood flow index. The basic principle utilizes the speckle phenomena, an intensity pattern produced by the mutual interference of a set of wave fronts, such as laser light [11–13]. The change in pattern mapped out by the erythrocyte movement in the ocular fundus can be analyzed to obtain the MBR, an indicator of the retinochoroidal blood flow [20]. LSFG has been used to aid in the diagnosis of various conditions, including glaucoma and retinal vein occlusion [20–22]. It has also been applied to the study of ocular microcirculation in neonates [23] and studies of changes in retinochoroidal blood flow with age [24–26]. Haga et al. reported on the utility of LSFG before and after CAS but with a small sample of seven cases [27].

In this study, we have evaluated the effectiveness of LSFG for assessing the ocular microcirculation before and after CAS of 40 lesions. We also investigated the correlation between the ocular microcirculation and the direction of the ocular artery blood flow by classifying the patients into antegrade and nonantegrade groups according to their LSFG findings before CAS. Our results determined that there was a significant increase in the MBR after CAS. Since LSFG may be affected by physiological conditions, systemic blood pressure and contralateral MBR were also measured during the procedure. Systemic blood pressure was significantly lower following surgery and the MBR did not show any significant change in the contralateral side, leading us to conclude that neither physiological nor individual differences affected the LSFG mapping. This heightened our anticipation that CAS could improve ocular microcirculation and effectively treat OIS.

We also evaluated the direction of ophthalmic artery blood flow before and after CAS. Development of the ophthalmic artery involves multiple anastomoses with both the internal and external carotid artery, along with regression of several primitive arteries [2, 3]. Multiple anastomoses are evident in the maxillary artery branch—an external carotid artery branch—and the distal branch of the ophthalmic artery [2, 3], often creating a retrograde blood flow stemming from the external carotid artery in advanced ICA stenosis and ICA occlusion. In our evaluation, comparison of the rate of constriction in the antegrade and nonantegrade groups found that there was a significantly higher rate of constriction in the nonantegrade group. Tsai et al. reported that reversed ophthalmic artery flow observed by ophthalmic artery Doppler waveform is a predictor for advanced ICA stenosis that results in poor functional outcomes [28]. In our investigation, all cases in the retrograde group had successfully recovered antegrade flow after CAS. Furthermore, the significant rise in the MBR after CAS in the retrograde group suggests that revascularization improves ocular microcirculation, thus simultaneously treating and reducing the risks of OIS. It also indicates a positive prognosis for the patient's visual function. These results illustrate the importance of blood flow directionality in the ophthalmic artery in advanced ICA stenosis patients. In cases where retrograde blood flow is found, evaluation of the ocular microcirculation by LSFG to determine the presence of OIS is beneficial, as it can ultimately lead to the preservation of visual function.

As the observation period in this study was short-term, investigation of visual function before and after the surgery was not considered. For future studies, it would be optimal to ascertain the mid- to long-term effects of improved ocular microcirculation by evaluating the visual field, visual acuity, and retinal sensitivity.

## 5. Conclusion

We found LSFG to be an effective technique for evaluation of the retinochoroidal blood flow before and after CAS. The significant rise in the MBR after CAS indicated an improvement in the ocular microcirculation. Patients presenting with retrograde flow are particularly at risk of poor visual outcomes; however, revascularization surgery can recover antegrade flow and yield a significant improvement in the ocular microcirculation. We propose the use of angiography before and after surgery to observe blood flow directionality and LSFG to evaluate the ocular microcirculation as a necessary and important step for the long-term preservation of the patient's visual function.

## Figures and Tables

**Table 1:** Average MBR and mean arterial pressure.

	Before CAS	After CAS	*P* value
Ipsilateral MBR	16.7 ± 4.8	18.5 ± 4.8	0.003
Contralateral MBR	17.7 ± 4.4	18.6 ± 4.0	0.22
MAP (mmHg)	102.3 ± 14.2	95.1 ± 12.4	<0.001

^*∗*^(*N* = 40).

^*∗∗*^MAP = 1/3 (systolic blood pressure – diastolic blood pressure) + diastolic blood pressure.

^*∗∗∗*^MAP, mean arterial pressure; SBP, systolic blood pressure; DBP, diastolic blood pressure.

**Table 2:** Patient characteristics.

	Antegrade	Nonantegrade	*P* value
Number of patients	22	18	
Age	78.3 ± 6.6	73.5 ± 6.2	0.02
Sex	M: 19 F: 3	M: 15 F: 3	0.57

PSV (cm/s)	323.0 ± 80.0	445.6 ± 95.1	0.002

Direction of OA flow after CAS			
Antegrade, *n* (%)	22 (100)	16 (88.9)	

MBR before CAS	17.9 ± 4.8	15.3 ± 4.7	0.052
MBR after CAS	19.1 ± 5.0	17.8 ± 4.7	0.57

^*∗*^Values are mean ± SD.

^*∗∗*^PSV, peak systolic velocity; OA, ophthalmic artery.
