# Corrigendum to “Relationship between the Direction of Ophthalmic Artery Blood Flow and Ocular Microcirculation before and after Carotid Artery Stenting”

**DOI:** 10.1155/2017/7861491

**Published:** 2017-11-12

**Authors:** Masashi Ishii, Morito Hayashi, Fumihiko Yagi, Kenichiro Sato, Goji Tomita, Satoshi Iwabuchi

**Affiliations:** ^1^Department of Neurosurgery, Toho University Ohashi Medical Center, Tokyo, Japan; ^2^Department of Ophthalmology, Toho University Ohashi Medical Center, Tokyo, Japan

In the article titled “Relationship between the Direction of Ophthalmic Artery Blood Flow and Ocular Microcirculation before and after Carotid Artery Stenting” [1], there was an error in Figure 6. For the nonantegrade group (*N* = 18), Figure 6 should show 18 lines, but showed 19 lines because one case from the antegrade group was incorrectly included. This error does not change the statistical results, because the calculation was done correctly on 18 cases.  Figure 6 should be corrected as follows:

Additionally, reference 27 was incorrectly quoted. Therefore, the text reading “Haga et al. reported on the utility of LSFG before and after CAS but with a small sample of seven cases [27]” should be changed to “Haga et al. reported on the utility of LSFG before and after CEA but with a small sample of five cases [27].”

## Figures and Tables

**Figure 1:**
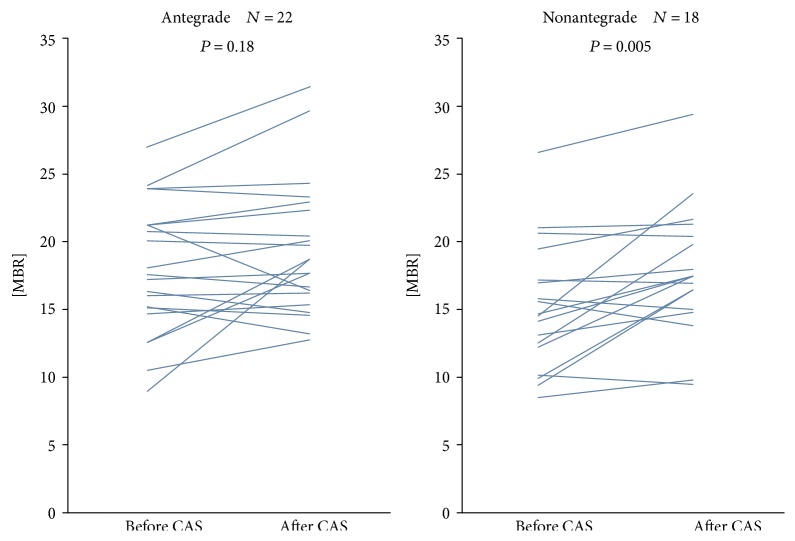
Change in the MBR before and after CAS in the antegrade and nonantegrade groups. There was the significant rise in the MBR in the nonantegrade group (*P* = 0.005).
